# Acupuncture combined with auricular acupressure for smoking cessation and its effects on tobacco dependence and smoking behavior among Hong Kong smokers: a multicenter pilot clinical study

**DOI:** 10.1186/s13020-022-00649-w

**Published:** 2022-08-09

**Authors:** Lai Fun Ho, Wai Kwan Ho, Ling Ling Wong, Sze Wan Chiu, Shuk Yu Tang, Chun Ming Wong, Kin San Chan, Chi Lan Lam, Min Chen, Kam Leung Chan, Guohua Lin, Bacon Fung-Leung Ng, Zhi Xiu Lin

**Affiliations:** 1 Chinese Medicine Services, Pok Oi Hospital, G/F, Shatin (Taiwai) Clinic, 2 Man Lai Road, Taiwai, Shatin, NT, Hong Kong, SAR China; 2 School of Chinese Medicine, Faculty of Medicine, The Chinese University of Hong Kong, Hong Kong, SAR China; 3 Hong Kong Institute of Integrative Medicine, Faculty of Medicine, The Chinese University of Hong Kong, Hong Kong, SAR China; 4 The First Affiliated Hospital, Guangzhou University of Chinese Medicine, Guangzhou, China; 5 Chinese Medicine Department, Hospital Authority, Hong Kong, SAR China

**Keywords:** Acupuncture, Auricular acupressure, Clinical trial, Smoking cessation, Tobacco dependence

## Abstract

**Background:**

Acupuncture combined with auricular acupressure has been used as a complementary and alternative treatment for smoking cessation in Hong Kong for over 10 years. This study aimed to investigate the success rates of smoking cessation posttreatment, and to evaluate treatment effects on tobacco dependence, smoking behavior, anxiety levels, and sleep disturbances between successful and unsuccessful quit smokers in Hong Kong.

**Methods:**

This prospective, multicenter clinical study conducted between September 2020 and February 2022 in Hong Kong was part of the Guangdong-Hong Kong-Macau Greater Bay Area project on smoking cessation. Thirty eligible current smokers (mean age 47.10 years; 40% female) were recruited and received a combination of standardized acupuncture and auricular acupressure treatments twice weekly for 8 weeks. The primary outcome was the success rate of smoking cessation at week 24. The secondary outcomes were the success rates of smoking cessation at weeks 8 and 16, exhaled carbon monoxide (CO) levels, and changes in scores on the Fagerström Test for Nicotine Dependence (FTND), Autonomy Over Smoking Scale (AUTOS), Hamilton Anxiety Rating Scale (HAM-A), Self-rating Anxiety Scale (SAS), and Pittsburgh Sleep Quality Index (PSQI). Adverse events were also recorded.

**Results:**

Of 30 eligible participants, 28 completed 6 or more treatment sessions; all completed follow-up assessments. At week 24, the success rate of smoking cessation was 46.67%. The successfully quit rates at weeks 8 and 16 were 36.67% and 43.33%, respectively. The overall change in mean FTND scores from baseline improved significantly from weeks 2 to 24 (*P* < 0.05), with the successful quit group showing significantly greater improvement between weeks 8 and 24 (*P* < 0.01). Compared with baseline values, there were significant reductions in mean AUTOS scores from weeks 6 to 24 (*P* < 0.001), with the successful quit group showing greater improvement at weeks 16 (*P* = 0.04) and 24 (*P* < 0.001). No significant changes were detected in exhaled CO levels or HAM-A, SAS, and PSQI scores. No study-related adverse events were observed.

**Conclusions:**

Acupuncture combined with auricular acupressure could be an effective alternative treatment for smoking cessation and reduction of tobacco dependence among Hong Kong smokers.

*Trial registration* Chinese Clinical Trial Registry, No. ChiCTR2000033650. Registered on June 7, 2020. http://www.chictr.org.cn/showproj.aspx?proj=54866

## Background

Tobacco smoking is a challenging public health problem worldwide. The global prevalence of smoking among people aged greater than 15 years was 17.5% in 2019 [1]. In Hong Kong, the Census and Statistics Department Survey Report for 2019 showed that cigarette smoking prevalence was 11.1% among those aged 15 years and older [2]. Smoking is a major cause of and associated with many preventable diseases and premature deaths internationally [1]. According to the Global Burden of Diseases, Injuries, and Risk Factors Study 2019, smoking was the second leading risk factor for attributable deaths, accounting for 8.71 million deaths worldwide [3].

Tobacco smoking increases the risk of contracting a wide range of diseases, including lung and heart diseases, chronic respiratory diseases, cancers, and diabetes, many of which are fatal. In general, there is a positive association between average daily cigarette consumption and the risk of most smoking-related diseases, and cigarette use can also harm non-smokers who are exposed to environmental tobacco smoke [4].

Quitting tobacco smoking has a profound effect on improving health and quality of life and significantly reduces the risk of tobacco-related diseases and death. Stopping smoking at any age is more beneficial than continuing to smoke. Reducing tobacco use is critical for reducing the burden of noncommunicable diseases, which account for 71% of deaths globally [1]. Many countries have adopted smoke-free legislative measures for tobacco control. In addition, various methods, including but not limited to pharmacotherapy, nicotine replacement therapy, and behavioral therapy, are available to help smokers achieve abstinence. Bupropion and varenicline are non-nicotine medications used for smoking cessation. Nicotine replacement therapy helps reduce the motivation to smoke and is accepted as an essential treatment for people who want to stop smoking. Numerous trials attempting to evaluate these treatments have resulted in different levels of evidence; however, no single best treatment method has been identified [5].

Acupuncture has been used for smoking cessation for years, with increasing research into acupuncture’s effect on nicotine dependence leading to more widespread acceptance. A previous clinical study found that acupuncture alleviates cue-induced cravings during the initial abstinence phase of smoking cessation through the regulation of brain activation in areas involved in attention, motivation, and reward [6]. A study exploring the neural mechanisms of acupuncture indicated that the immediate effects of acupuncture on smoking cravings were significant and identified the anterior cingulate cortex, insula, prefrontal cortex, visual cortex, and cerebellum as the key brain areas involved in the response [7]. Previous clinical studies have also shown that acupuncture is effective for smoking cessation [8, 9], its treatment effect is noninferior to that of nicotine replacement therapy [9], and said effect may last for at least 5 years [10]. Acupuncture is also safe and can relieve withdrawal symptoms [8] and reduce daily cigarette consumption [11, 12].

Auricular acupressure works by continuously stimulating acupoints to regulate and relieve disease symptoms; consequently, auricular acupressure has also been adopted as a simple and effective noninvasive intervention to help quit smoking, as it may help relieve withdrawal symptoms and decrease levels of exhaled carbon monoxide (CO) [13]. The results from a previous systematic review and meta-analysis suggested that ear acupressure is beneficial to achieving smoking cessation and could be a clinical alternative to established smoking cessation interventions [14]. However, this result was based on data without biochemical confirmation.

In view of the above, acupuncture, auricular acupressure, or a combination of both could be effective treatments for smoking cessation; however, their efficacy remains controversial, and high-quality scientific evidence is currently lacking [15]. An earlier systematic review and Bayesian network meta-analysis showed that the probability rankings of auricular acupressure and acupuncture plus auricular acupressure were superior to other interventions in smoking cessation [16]. Nonetheless, substantial uncertainty remains as other previous studies showed conflicting results, and no firm conclusions can yet be made regarding the effectiveness of acupuncture as a single treatment for smoking cessation.

In Hong Kong, a free community-based smoking cessation service utilizing body acupuncture and auricular acupressure has been provided since April 2010, with favorable results [8, 12]. This study aimed to investigate the success rates of smoking cessation among Hong Kong smokers who underwent 8 weeks of acupuncture combined with auricular acupressure treatments. In addition, we evaluated changes in tobacco dependence, smoking behavior, anxiety levels, and sleep disturbances between successful and unsuccessful quit smokers.

## Methods

### Study design and setting

This prospective, multicenter, open-label clinical study was conducted between September 24, 2020, and February 22, 2022. The trial sites included Pok Oi Hospital‒The Chinese University of Hong Kong Chinese Medicine Clinic cum Training and Research Centre (Shatin District), and two community-based mobile clinics. The trial consisted of 8 weeks of treatment and 16 weeks of follow-up (an 8-week treatment period with follow-up at weeks 16 and 24). The trial was approved by the Joint Chinese University of Hong Kong‒New Territories East Cluster Clinical Research Ethics Committee (No. 2020.116) and registered in the Chinese Clinical Trial Registry (No. ChiCTR2000033650). The trial adhered to the Declaration of Helsinki and Good Clinical Practice guidelines. The study was conducted in accordance with the STandards for Reporting Interventions in Clinical Trials of Acupuncture recommendations [17]. All participants provided written informed consent prior to participation. Treatments were provided free, and no subjects were paid for their participation.

### Participants

Current smokers who wanted to quit smoking were recruited and underwent eligibility assessment by cross-referral from all Chinese medicine service units under the management of Pok Oi Hospital (covering all districts in Hong Kong) between September 2020 and August 2021.

Eligible participants had to meet the following inclusion criteria: (1) voluntarily quit smoking; (2) aged 18‒65 years; (3) smoked for ≥ 1 year; (4) consumed ≥ 20 cigarettes per day in the previous year; (5) positive salivary cotinine test; (6) nicotine dependence score (Fagerström Test for Nicotine Dependence, FTND [18]) of ≥ 4 points; (7) provided written informed consent and volunteered to participate; and (8) undergone a washout period of more than 1 month if other smoking cessation treatments were previously conducted.

Participants were excluded if they: (1) had severe and unstable cardiac, pulmonary, cerebral, or hematologic diseases or diabetes with other complications; (2) had mental illness or drug abuse; (3) had apoplexy or other nervous system diseases; (4) had unknown diseases; (5) had blood coagulation disturbances or were undergoing treatment with anticoagulant drugs; (6) had moderate or severe liver or kidney impairment; (7) were pregnant; or (8) had a history of smoking cessation treatments, such as acupuncture, auricular acupressure, or nicotine replacement therapy, within the previous month.

After eligibility assessment by the research team, the participants were enrolled and underwent baseline pretreatment evaluations. Routine biochemical and hematological tests and electrocardiography were performed before treatment administration.

### Intervention

All participants received standardized acupuncture and auricular acupressure treatments twice weekly for 8 consecutive weeks. The treatments were performed by Hong Kong Registered Chinese Medicine Practitioners who had over 3 years of practice experience and received training to execute standard protocol procedures prior to study initiation. The treatment protocol was developed based on the neural mechanism of acupuncture [6, 7], previous clinical studies [19, 20], and the clinical experience of acupuncture experts.

For acupuncture treatments, the acupoints Baihui (GV20), Yintang (GV29), bilateral Lieque (LU7), and bilateral Hegu (LI4) [21, 22] were used based on the methodology of an earlier smoking cessation study [20]. During each treatment session, the participants were in a supine position exposing the acupoints. After skin disinfection, sterile, single-use acupuncture needles (0.25 mm × 25/40 mm; Suzhou Medical Appliance Factory, Suzhou, China) were inserted obliquely into GV20 (depth, 0.8–1.0 cun) and bilateral LU7 (depth, 0.5–1.0 cun until it reached Yangxi [LI5]), inserted transversely into GV29 (depth, 0.8 cun), and inserted perpendicularly into bilateral LI4 (depth, 0.5–0.8 cun). The needles were manipulated using the lifting and thrusting method [23] until deqi sensation [24] was achieved for each acupoint whenever possible, and retained in situ for 30 min before removal. During retention, the needles were manipulated by lifting and thrusting 20 times every 10 min.

Auricular acupressure was administered unilaterally, alternating between the left and right ear in each treatment session. The auricular acupoints used were Shenmen (TF_4_), Fei (CO_14_), Wei (CO_4_), Neifenmi (CO_18_), Pizhixia (AT_4_), Jiaogan (AH_6a_), and Kou (CO_1_) [25]. After skin disinfection, Vaccariae seeds (Wang Bu Liu Xing) 2 mm in diameter were adhered with adhesive tape to the surface of the 7 auricular acupoints and maintained for 3 days. Participants were instructed to press each auricular acupoint via the central plastered seed 3–5 times per day for 30–60 s.

During the study period, the research team advised each participant to avoid any other treatments for smoking cessation but to continue medications for diseases such as hypertension, diabetes, hyperlipidemia, and coronary heart disease.

### Outcome measures

Evaluations in this study were performed at baseline and at weeks 2, 4, 6, and 8 after the start of treatment and again at weeks 16 and 24 when follow-up was performed.

The primary outcome measure was the success rate of smoking cessation at week 24. Success was defined as self-reported quitting and verified by an exhaled CO level < 10 parts per million (ppm) within 24 h of self-reported abstinence, measured using CO Check Pro (MD Diagnostics Ltd, Chatham, UK), and a negative test result for salivary cotinine, measured using the Oral Fluid Cotinine Test Mini Cube (TestCountry, San Diego, CA, USA).

Secondary outcomes were the success rates of smoking cessation at weeks 8 and 16, exhaled CO levels at each evaluation time point, efficacy indicators of the FTND and Autonomy Over Smoking Scale (AUTOS) at each evaluation time point, and scale evaluations of the Hamilton Anxiety Rating Scale (HAM-A), Self-rating Anxiety Scale (SAS), and Pittsburgh Sleep Quality Index (PSQI) at each evaluation time point. The FTND (score, 0–10) is a validated 6-item instrument devised to evaluate the number and strength of cigarettes smoked as well as smoking behavior, with higher scores indicating higher levels of nicotine dependence [18]. AUTOS (score, 0–36) is a validated 12-item theory-based instrument with three subscales to assess withdrawal symptoms, psychological dependence, and cue-induced craving [26, 27]. HAM-A (score, 0–56) is a validated 14-item (symptom-defined elements) clinician-rated instrument for quantifying anxiety symptoms, with higher scores indicating greater symptom severity [28, 29]. The SAS (index, 25–100) is a validated 20-item rating instrument for anxiety disorders, with higher scores indicating greater anxiety [30]. PSQI (score, 0–21) is a validated 19-item self-rated questionnaire for assessing sleep quality and disturbances, with higher scores indicating poorer sleep quality [31]. Possible predictors for smoking cessation were evaluated, and all adverse events or reactions were monitored, appropriately managed, and recorded throughout the study period.

### Statistical analysis

This study was part of the Guangdong-Hong Kong-Macau Greater Bay Area Smoking Cessation Project, and the Hong Kong site was assigned to recruit 30 subjects. Therefore, no additional formal sample size calculations were performed. This sample size is similar to that recommended by previous research on sample size determination for pilot trials [32].

We used the statistical package IBM SPSS Statistics version 27.0 (IBM Corp, Armonk, NY, USA) for conducting statistical analyses, and all participants’ data were included. Analyses were performed by a statistician not involved in treatment provision or data collection. All statistical tests were two-sided, with a significance level of 0.05.

To analyze the data, appropriate parametric or nonparametric statistical tests were used in accordance with the nature of the data. Baseline participant characteristics were reported as mean and standard deviation for continuous variables and as count and percentage for categorical variables.

To investigate changes in the mean scores for all continuous outcome variables from baseline to week 24, linear mixed-effects model (LMM) analyses were conducted. The fundamental assumptions of the LMM (including normality, validity of the model, and independence of data points) were tested to ensure the accuracy of the test results.

The overall and individual group changes for each continuous outcome from baseline across all measurement points were analyzed using LMM analyses. In addition, LMM analyses were performed to compare treatment effects between the successful and unsuccessful quit groups across all study time points from baseline to week 24 (group-by-time interaction effects). Post-hoc between-group and within-group differences were computed using the Bonferroni adjustment for multiple comparisons (six time points).

Furthermore, binary logistic regression was adopted to analyse the relationship between a series of baseline characteristics that could act as possible predictors and the success rate of smoking cessation at week 24 and was reported as odds ratio and 95% confidence interval (OR and 95% CI). Adverse events and additional information were descriptively analyzed and reported.

## Results

### Participant flow and baseline characteristics

**Fig. 1:**
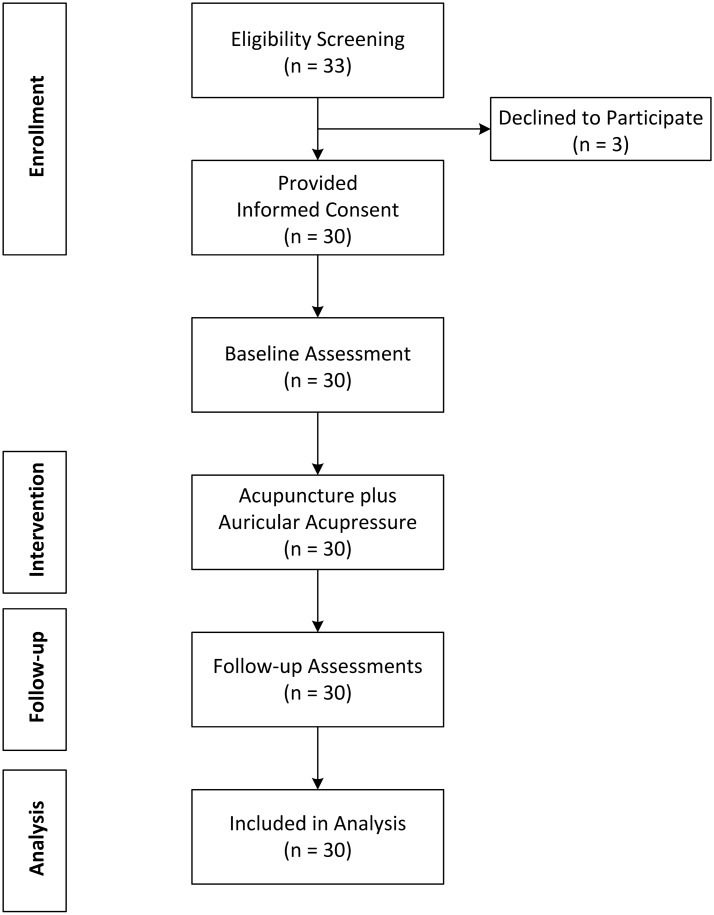
Study flowchart

From September 2020 to August 2021, a total of 33 current smokers who expressed interest in the study were screened. Of these 33 smokers, 3 declined participation before the start of treatment. The remaining 30 participants were enrolled and treated; none were lost to follow-up. Of these 30 participants, 15 completed all 16 treatment sessions, and only 2 received less than 6 treatment sessions. All pretreatment salivary cotinine test results were positive. The study flowchart is shown in Fig. 1.

The demographic and clinical characteristics of the participants are presented in Table 1. All participants were Chinese, 40.00% were female, and 76.67% were aged 40 years and above. The mean age was 47.10 ± 10.74 years, and most participants were between 40 and 49 years old (43.33%). The mean duration of smoking was 24.10 ± 11.43 years, and 70.00% of the participants smoked at least 10 cigarettes per day. In addition, 46.67% of participants had tried to quit smoking in the past. The mean scores for FTND and AUTOS were 5.06 ± 1.87 and 19.33 ± 7.99, respectively. Moreover, we presented the baseline data of participants by categorizing them into successful and unsuccessful quit smokers (based on smoking cessation at week 24). The baseline characteristics of the two groups were similar, except that those who successfully quit smoking had a lower level of exhaled CO before treatment.

**Table 1:** Baseline participant characteristics

Characteristic		Total (n = 30)	Successful Quit (n = 14)	Unsuccessful Quit (n = 16)		*P* value
Age, y		47.10 ± 10.74	49.50 ± 13.11	45.00 ± 8.01		0.26
Sex						0.76
Female		12 (40.00)	6 (42.86)	6 (37.50)		
Male		18 (60.00)	8 (57.14)	10 (62.50)		
Body mass index, kg/m^2^		25.10 ± 3.69	25.24 ± 3.08	24.98 ± 4.26		0.85
Marital status						0.76
Married		18 (60.00)	8 (57.14)	10 (62.50)		
Single or divorced		12 (40.00)	6 (42.86)	6 (37.50)		
Education level						0.35
Secondary		26 (86.67)	13 (92.86)	13 (81.25)		
Post-secondary or higher		4 (13.33)	1 (7.14)	3 (18.75)		
Occupation						0.51
Currently employed		19 (63.33)	8 (57.14)	11 (68.75)		
Unemployed/retired/other		11 (36.67)	6 (42.86)	5 (31.25)		
Family type						0.58
Two-parent family		23 (76.67)	11 (78.57)	12 (75.00)		
Single-parent family		7 (23.33)	3 (21.43)	4 (25.00)		
Living condition						0.75
Living alone		3 (10.00)	2 (14.29)	1 (6.25)		
With family members		25 (83.33)	11 (78.57)	14 (87.50)		
With others		2 (6.67)	1 (7.14)	1 (6.25)		
Smoking duration		24.10 ± 11.43	22.85 ± 14.81	25.18 ± 7.73		0.60
Daily cigarette consumption*		14.41 ± 7.16	13.28 ± 8.18	15.46 ± 6.16		0.42
Previous quit attempts						0.06
None		16 (53.33)	10 (71.43)	6 (37.50)		
≥ 1		14 (46.67)	4 (28.57)	10 (62.50)		
Reason to quit smoking						0.26
Own health		21 (70.00)	9 (64.29)	12 (75.00)		
Family suggestion		3 (10.00)	1 (7.14)	2 (12.50)		
Medical suggestion		1 (3.33)	0 (0.00)	1 (6.25)		
Other reasons		5 (16.67)	4 (28.57)	1 (6.25)		
Exhaled CO Level (ppm)						0.001
< 10 ppm		18 (60.00)	13 (92.86)	5 (31.25)		
≥ 10 ppm		12 (40.00)	1 (7.14)	11 (68.75)		
FTND		5.06 ± 1.87	5.14 ± 2.17	5.00 ± 1.63		0.83
AUTOS						
Withdrawal symptoms		6.33 ± 3.20	6.78 ± 2.75	5.93 ± 3.60		0.48
Psychological dependence		5.83 ± 3.20	6.35 ± 3.20	5.37 ± 3.24		0.41
Cue-induced craving		7.16 ± 2.50	7.85 ± 2.31	6.56 ± 2.58		0.16
Total		19.33 ± 7.99	21.00 ± 7.53	17.87 ± 8.34		0.29
HAM-A		16.06 ± 9.02	18.85 ± 9.33	13.62 ± 8.26		0.11
SAS		50.80 ± 7.82	50.92 ± 8.84	50.68 ± 7.10		0.93
PSQI		7.43 ± 4.05	7.35 ± 4.03	7.50 ± 4.21		0.92

Data are expressed as mean ± standard deviation or as number (%)

CO, carbon monoxide; ppm, parts per million; FTND, Fagerström Test for Nicotine Dependence; AUTOS, Autonomy Over Smoking Scale; HAM-A, Hamilton Anxiety Rating Scale; SAS, Self-rating Anxiety Scale; PSQI, Pittsburgh Sleep Quality Index

*One participant in the unsuccessful quit group used electronic cigarettes

### Primary outcome

**Fig. 2:**
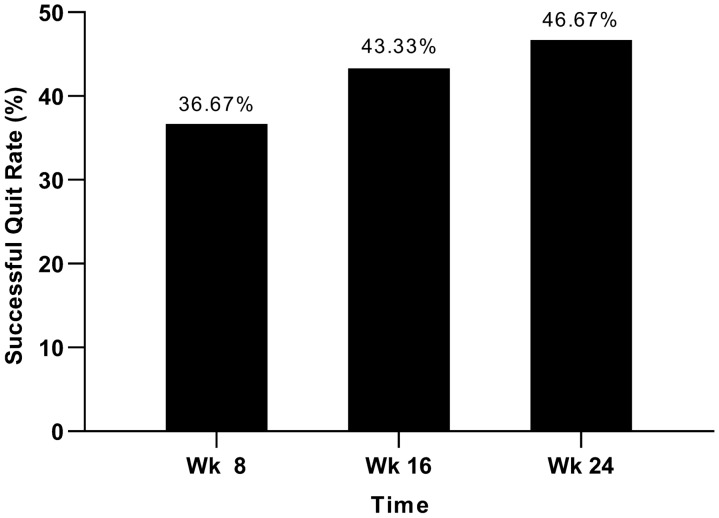
Successful quit rates at different time points. Wk, Week

Regarding the primary outcome, the success rate of smoking cessation at week 24 was 46.67% (Fig. 2). Thirteen out of the 14 participants who successfully quit smoking attended 6 or more treatment sessions. Among those who did not quit smoking (n = 16), 11 (68.75%) reported a reduction in the number of cigarettes smoked per day at week 24.

### Secondary outcomes

The success rates for smoking cessation at weeks 8 and 16 were 36.67% and 43.33%, respectively (Fig. 2). Among those who did not quit smoking, 63.16% and 70.59% reported a reduction in daily cigarette consumption at weeks 8 and 16, respectively.

**Fig. 3:**
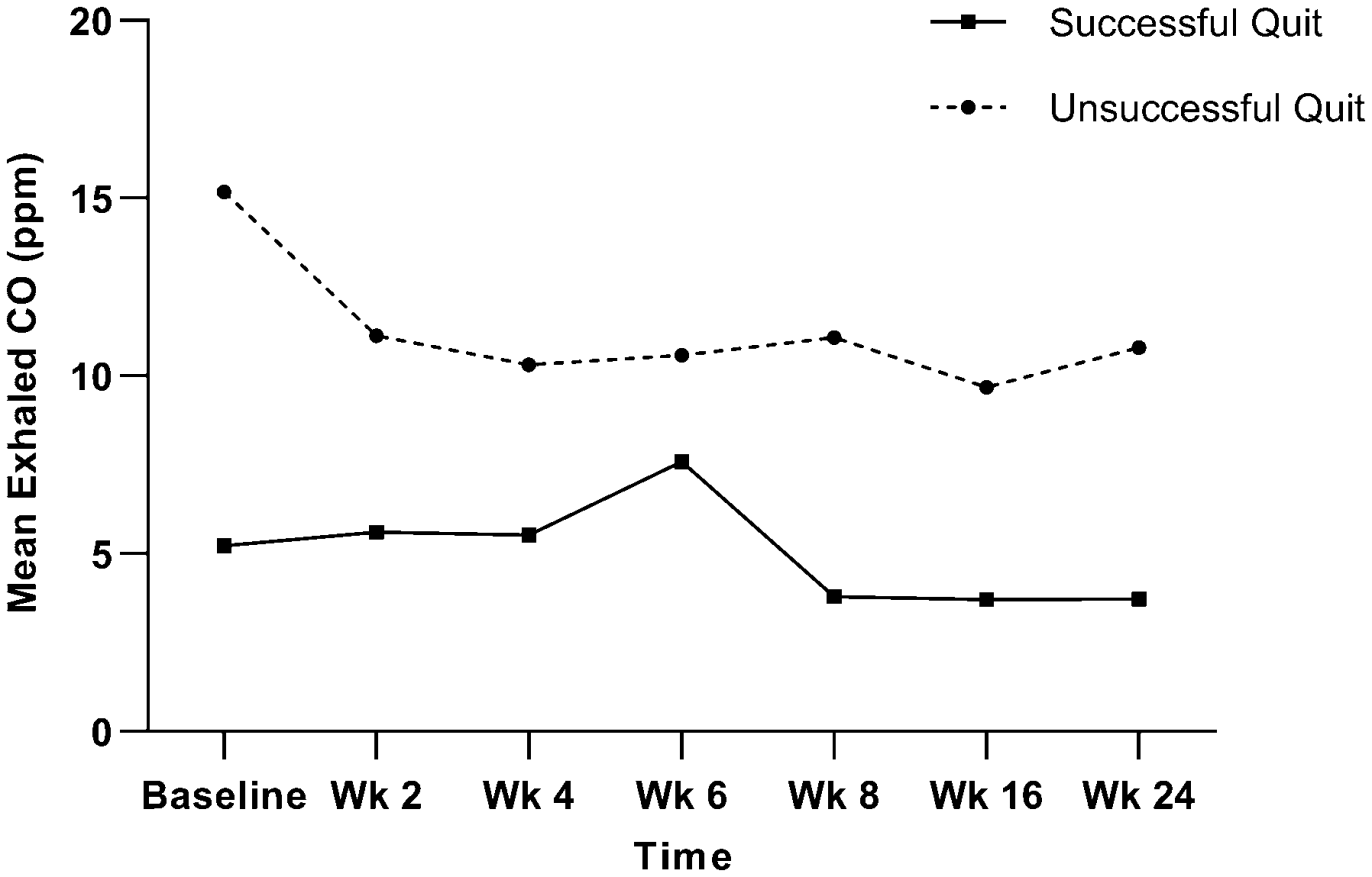
Changes in mean exhaled CO levels over time. CO, carbon monoxide; ppm, parts per million; Wk, Week

Overall, the mean exhaled CO levels for all participants showed a gradual reduction over time; however, no significant changes were detected (*P* > 0.05). The mean exhaled CO levels at each time point for the successful and unsuccessful quit groups are illustrated in Fig. 3, and no significant changes over time were found in either group (*P* > 0.05).

**Fig. 4:**
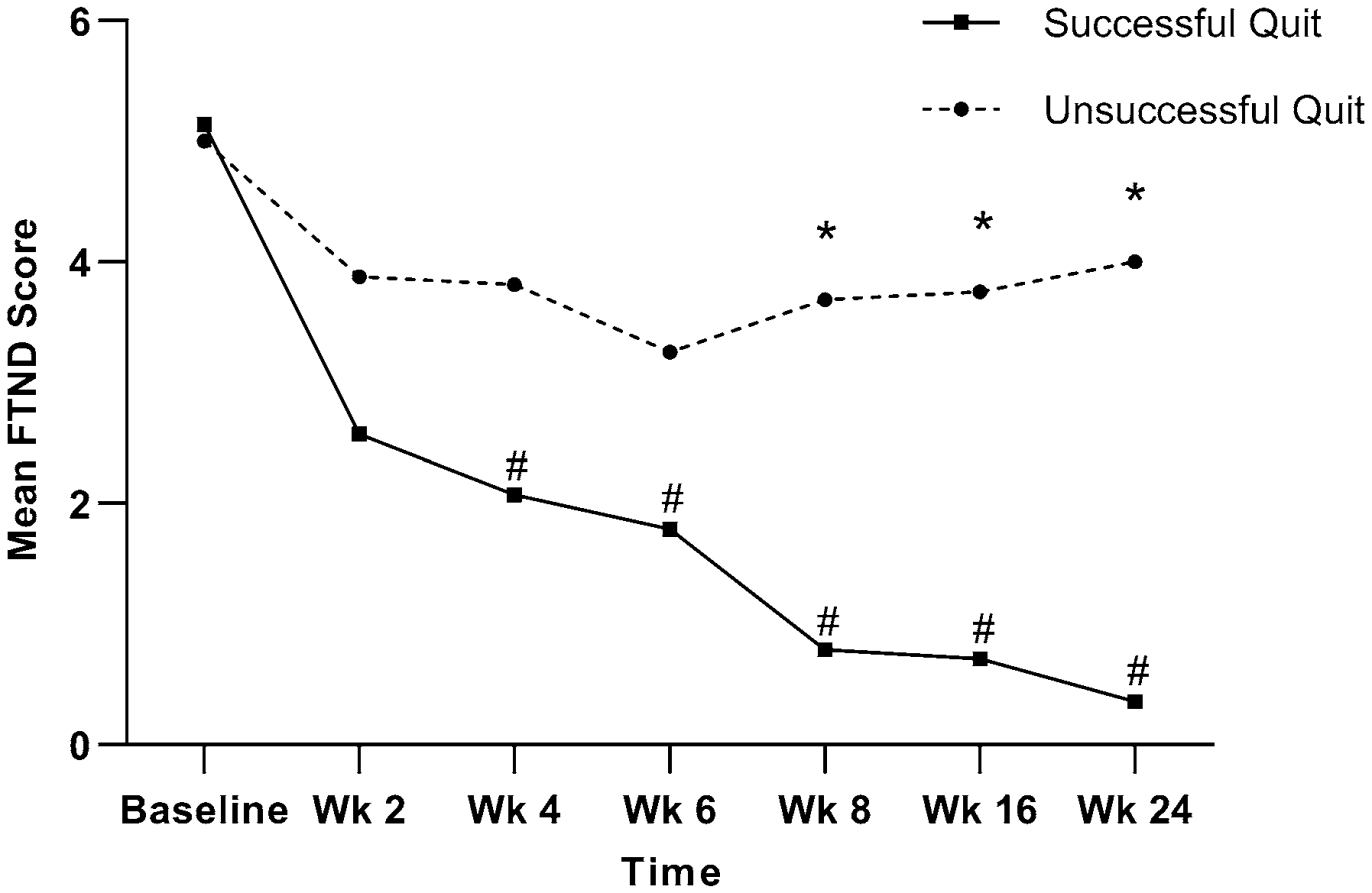
Changes in mean FTND scores over time. FTND, Fagerström Test for Nicotine Dependence; Wk, Week. * denotes a significant between-group difference; # denotes a significant within-group difference compared with the pretreatment value

The mean FTND scores for both the successful and unsuccessful quit groups at each time point are shown in Fig. 4. LMM analysis indicated significant effects of group (*P* < 0.001), time (*P* < 0.001), and group-by-time interaction (*P* = 0.003) for FTND. Significant between-group differences were identified between weeks 8 and 24, with the successful quit group having a significantly greater reduction in mean FTND scores. In the successful quit group, the FTND scores from weeks 4 to 24 were significantly lower than those at baseline; however, these improvements were not observed in the unsuccessful quit group (Table 2).

**Table 2:** Change from baseline for secondary outcome measures at different time points

Outcome	Total	*P* value^a^	Successful Quit (n = 14)	Unsuccessful Quit (n = 16)	*P* value^b^
FTND					
Week 2	− 0.80 (− 3.51 to − 0.08)	0.03	− 2.57 (− 5.44 to 0.30)	− 1.12 (− 3.27 to 1.02)	0.18
Week 4	− 2.06 (− 3.77 to − 0.35)	0.006	− 3.07 (− 6.01 to − 0.12)^*^	− 1.18 (− 3.17 to 0.79)	0.06
Week 6	− 2.50 (− 4.16 to − 0.84)	< 0.001	− 3.35 (− 6.21 to − 0.50)^*^	− 1.75 (− 3.70 to 0.20)	0.08
Week 8	− 2.73 (− 4.49 to − 0.97)	< 0.001	− 4.35 (− 6.98 to − 1.72)^‡^	− 1.31 (− 3.48 to 0.85)	0.006
Week 16	− 2.73 (− 4.51 to − 0.95)	< 0.001	− 4.42 (− 7.02 to − 1.83)^‡^	− 1.25 (− 3.44 to 0.94)	0.004
Week 24	− 2.76 (− 4.48 to − 1.05)	< 0.001	− 4.78 (− 7.04 to − 2.52)^‡^	− 1.00 (− 3.05 to 1.05)	< 0.001
AUTOS					
Week 2	− 3.30 (− 9.83 to 3.23)	1.00	− 4.85 (− 14.43 to 4.72)	− 1.93 (− 11.18 to 7.31)	0.90
Week 4	− 5.83 (− 12.22 to 0.55)	0.11	− 9.07 (− 18.80 to 0.65)	− 3.00 (− 11.74 to 5.74)	0.10
Week 6	− 8.93 (− 14.82 to − 3.04)	< 0.001	− 12.14 (− 21.65 to − 2.62)^†^	− 6.12 (− 13.62 to 1.37)	0.24
Week 8	− 10.73 (− 16.55 to − 4.91)	< 0.001	− 14.00 (− 23.48 to − 4.51)^†^	− 7.87 (− 15.54 to − 0.20)^*^	0.46
Week 16	− 12.56 (− 18.10 to − 7.02)	< 0.001	− 17.50 (− 25.99 to − 9.00)^‡^	− 8.25 (− 15.71 to − 0.78)^*^	0.04
Week 24	− 13.03 (− 19.10 to − 6.96)	< 0.001	− 20.28 (− 27.89 to − 12.67)^‡^	− 6.68 (− 15.09 to 1.72)	< 0.001
HAM− A					
Week 2	− 0.86 (− 8.64 to 6.91)	1.00	− 2.00 (− 13.48 to 9.48)	0.12 (− 10.29 to 10.54)	0.40
Week 4	− 4.13 (− 11.69 to 3.42)	1.00	− 5.78 (− 17.48 to 5.91)	− 2.68 (− 12.35 to 6.97)	0.60
Week 6	− 6.20 (− 13.54 to 1.14)	0.20	− 7.14 (− 19.51 to 5.23)	− 5.37 (− 13.85 to 3.10)	0.55
Week 8	− 6.53 (− 13.82 to 0.75)	0.12	− 8.14 (− 20.17 to 3.89)	− 5.12 (− 14.00 to 3.75)	0.80
Week 16	− 4.96 (− 12.13 to 2.20)	0.66	− 6.07 (− 17.98 to 5.84)	− 4.00 (− 12.62 to 4.62)	0.36
Week 24	− 5.80 (− 13.08 to 1.48)	0.29	− 9.14 (− 20.44 to 2.15)	− 2.87 (− 12.80 to 7.05)	0.97
SAS					
Week 2	− 2.40 (− 8.61 to 3.81)	1.00	− 1.00 (− 11.45 to 9.45)	− 3.62 (− 11.52 to 4.27)	0.23
Week 4	− 4.16 (− 9.97 to 1.64)	0.55	− 3.28 (− 13.06 to 6.48)	− 4.93 (− 12.41 to 2.54)	0.26
Week 6	− 3.60 (− 8.82 to 1.62)	0.66	− 3.21 (− 12.43 to 6.01)	− 3.93 (− 10.54 to 2.67)	0.47
Week 8	− 5.03 (− 10.95 to 0.89)	0.19	− 5.57 (− 16.06 to 4.92)	− 4.56 (− 11.92 to 2.79)	0.86
Week 16	− 3.66 (− 8.99 to 1.65)	0.67	− 3.00 (− 12.28 to 6.28)	− 4.25 (− 11.06 to 2.56)	0.27
Week 24	− 2.33 (− 7.58 to 2.91)	1.00	− 1.71 (− 10.77 to 7.34)	− 2.87 (− 9.71 to 3.96)	0.37
PSQI					
Week 2	− 0.80 (− 3.79 to 2.19)	1.00	− 1.00 (− 5.66 to 3.66)	− 0.62 (− 4.70 to 3.45)	0.64
Week 4	− 1.26 (− 4.43 to 1.90)	1.00	− 1.57 (− 6.56 to 3.41)	− 1.00 (− 5.38 to 3.38)	0.52
Week 6	− 1.53 (− 4.69 to 1.62)	1.00	− 1.85 (− 6.76 to 3.05)	− 1.25 (− 5.62 to 3.12)	0.44
Week 8	− 1.60 (− 4.68 to 1.48)	1.00	− 1.85 (− 6.61 to 2.89)	− 1.37 (− 5.67 to 2.92)	0.41
Week 16	− 1.53 (− 4.75 to 1.68)	1.00	− 2.00 (− 6.91 to 2.91)	− 1.12 (− 5.70 to 3.45)	0.45
Week 24	− 1.66 (− 5.06 to 1.73)	1.00	− 2.85 (− 7.64 to 1.93)	− 0.62 (− 5.61 to 4.36)	0.14

Data are presented as estimated mean (95% CI) from the linear mixed-effects model

FTND, Fagerström Test for Nicotine Dependence; AUTOS, Autonomy Over Smoking Scale; HAM-A, Hamilton Anxiety Rating Scale; SAS, Self-rating Anxiety Scale; PSQI, Pittsburgh Sleep Quality Index

^a^*P* values were calculated using a linear mixed-effects model to illustrate pre- and posttreatment differences

^b^*P* values were calculated using a linear mixed-effects model to illustrate between-group differences

^*^P < 0.05, compared with pretreatment within-group

^†^*P* < 0.01, compared with pretreatment within-group

^‡^*P* < 0.001, compared with pretreatment within-group

**Fig. 5:**
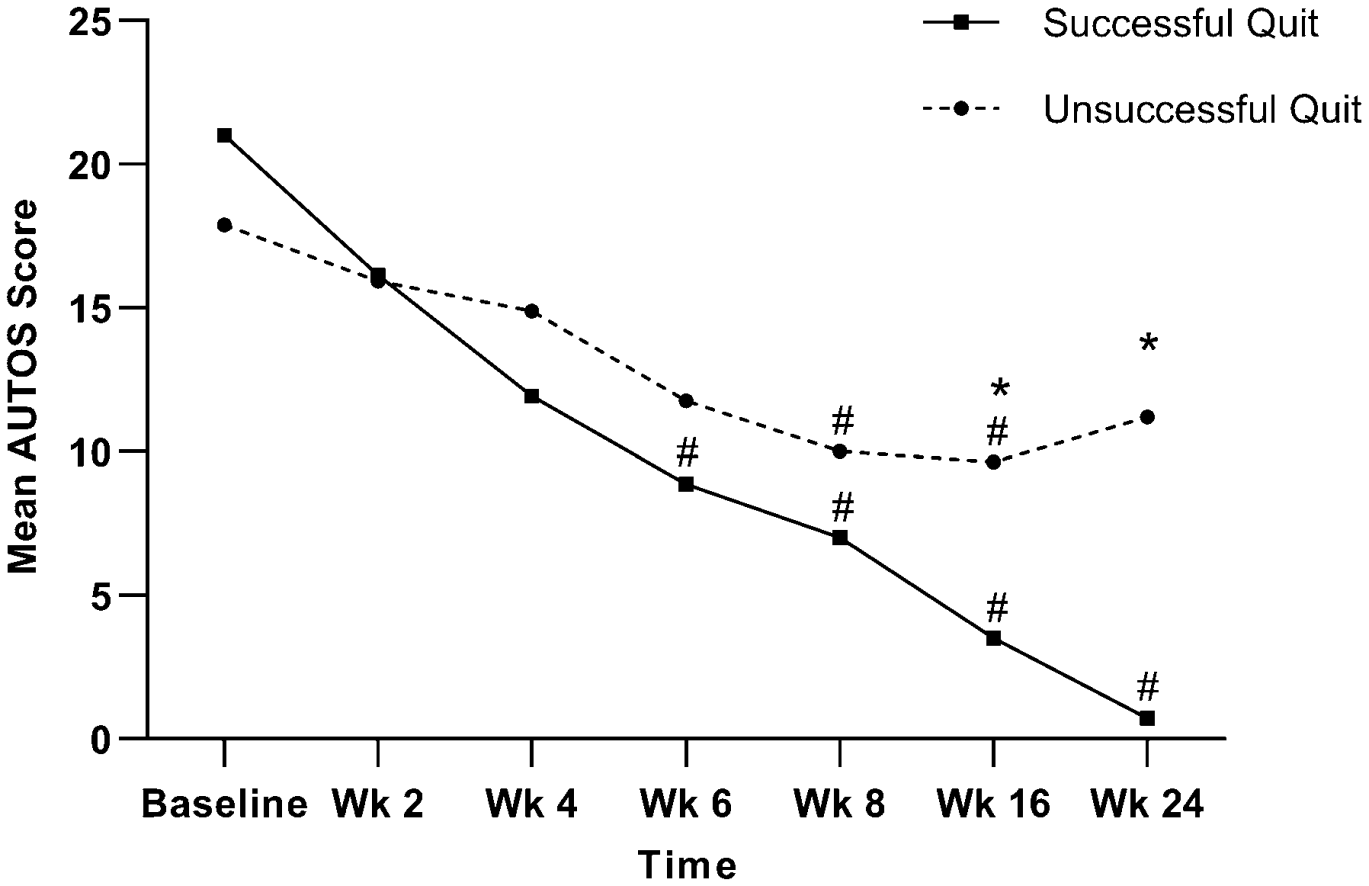
Changes in mean AUTOS scores over time. AUTOS, Autonomy Over Smoking Scale; Wk, Week. * denotes a significant between-group difference; # denotes a significant within-group difference compared with the pretreatment value

The mean AUTOS scores at different time points for the successful and unsuccessful quit groups are shown in Fig. 5. LMM analysis of changes in the mean AUTOS scores between both groups over time revealed significant effects of group (*P* = 0.012), time (*P* < 0.001), and group-by-time interaction (*P* = 0.002). Post-hoc between-group comparisons showed significantly greater improvement in mean AUTOS scores in the successful quit group at weeks 16 (*P* = 0.04, Table 2) and 24 (*P* < 0.001, Table 2). Relative to the baseline score, the mean AUTOS scores at weeks 6 to 24 in the successful quit group and at weeks 8 and 16 in the unsuccessful quit group exhibited significant improvements (Table 2).

Improvements were detected in HAM-A, SAS, and PSQI scores for both successful and unsuccessful quit groups over time, but their changes were not significant (all *P* > 0.05, Table 2). Moreover, between-group comparisons showed no significant differences in HAM-A, SAS, or PSQI scores across all study time points (all *P* > 0.05, Table 2).

In addition, binary regression analysis revealed that the exhaled CO level was a possible factor impacting smoking cessation success at week 24. Participants with higher exhaled CO levels at baseline were less likely to be successful in quitting smoking (OR = 0.72; 95% CI: 0.56‒0.92, *P* < 0.01).Throughout the study period, no study-related adverse events were reported.

## Discussion

Smoking is an important global public health problem, and many countries have taken numerous measures to address it. Various therapeutic approaches, including acupuncture, have been used to help people quit smoking. In Hong Kong, a free community-based smoking cessation service combining acupuncture and auricular acupressure has been provided since 2010, with positive results [8, 12, 33]. Our findings from the Hong Kong site in this Guangdong-Hong Kong-Macau Greater Bay Area project provide updated and additional evidence on the effects and safety of combined acupuncture and auricular acupressure for smoking cessation.

### Main findings

The 30 participants involved in this clinical trial were moderate to heavy smokers living in Hong Kong. Our study showed that the successful quit rate improved gradually from weeks 8 to 23. After combined acupuncture and auricular acupressure treatments, the success rates of smoking cessation achieved at weeks 8 (posttreatment) and 24 (follow-up period) were 36.67% and 46.67%, respectively. Among those who did not quit smoking, 63.16% and 68.75% reductions in cigarette consumption were recorded at weeks 8 and 24, respectively.

Regarding efficacy scales, the FTND scores in the successful quit group were significantly decreased from weeks 4 to 24 when compared to baseline; however, these improvements were not observed in the unsuccessful quit group. As for AUTOS, the mean scores at weeks 6–24 in the successful quit group and at weeks 8 and 16 in the unsuccessful quit group were significantly improved relative to the baseline.

No significant improvements were detected over time in exhaled CO levels or in HAM-A, SAS, and PSQI scores for both the successful and unsuccessful quit groups (all *P* > 0.05). Finally, no study-related adverse events were observed.

### Comparisons with previous studies

In general, our findings on abstinence rates were consistent with the previous multicenter observational study on acupuncture combined with auricular acupressure from Hong Kong (34.01% at week 8) [12] and the previous randomized controlled trial on acupuncture from China (36.00% at week 8 and 43.00% at week 24) [9]. Our study’s smoking cessation rates were noninferior to 8-week nicotine replacement therapy as reported in an earlier clinical trial in China (46.00% at week 8 and 44.00% at week 24). The higher abstinence rates recorded in our study may be because most of our participants possessed lower baseline exhaled CO levels. In a previous cohort study, successful quit smokers with 1-year abstinence exhibited lower levels of exhaled CO on day 8 of a 22-day intervention program than their unsuccessful counterparts [34]. Smoking cessation requires a strong will and a determined attempt to stop for most smokers. The careful and intense follow-up schedule employed in our study may have provided continuous support to smokers aiming to achieve smoking cessation.

Regarding FTND, a previous clinical trial reported improvements in mean scores over time, from week 8 (posttreatment) to week 24 (follow-up), for both acupuncture and auricular point pressing [9]. Our results indicated a greater improvement in FTND scores over time, which revealed that acupuncture combined with acupressure might have an effect on nicotine dependence. Further randomized controlled trials are warrented to confirm these findings.

Similar to previous study findings, the HAM-A scores in our study did not show any significant changes over time after treatment [35]. Moreover, SAS scores in our study declined over time, as was similarly observed in a previous clinical trial [36]. However, our results did not detect any significant reduction.

### Study strengths and limitations

The major aim of our study was to investigate the success rates of smoking cessation posttreatment. It has been reported that receiving sufficient and qualified acupuncture is a leading factor for short-term smoking cessation [33]. The more acupuncture treatment received, the greater the possibility of quitting tobacco dependence with acupuncture [8, 33]. A previous clinical trial in Norway reported that six acupuncture sessions might help motivate smokers to quit smoking completely, and the effect might last for five years [10]. In addition, another earlier study in Hong Kong demonstrated that 6 or more sessions of acupuncture were strongly associated with a higher chance of successful quitting [8]. In our study, over 90% of participants received 6 or more treatments, which may be one of the factors contributing to our higher rate of successful smoking cessation.

Moreover, measuring exhaled CO levels may provide an immediate, noninvasive method for assessing smoking status [37], and cotinine is the best indicator of tobacco smoke exposure [38]. Our study’s use of self-reported smoking cessation confirmed by exhaled CO levels and cotinine test results ensured objectivity and correctness and reduced the possibility of false claims.

Furthermore, this study’s use of validated and reliable measurement tools increased the reliability of the reported outcomes. FTND is a convenient and valid self-reported measure of nicotine dependency, and loss of autonomy is a good predictor of success at smoking cessation [26]. The improved FTND and AUTOS scores reported in our results showed the positive therapeutic effects of acupuncture on tobacco dependence in smokers. Furthermore, HAM-A is a reliable and valid anxiety evaluation questionnaire, and SAS is a rating instrument for measuring anxiety disorders, while PSQI provides a standardized, quantitative measure of sleep quality. Although no significant changes were found in these scores, our study still provided preliminary insight into the effects of combined acupuncture and auricular acupressure on smokers’ anxiety status and sleep quality.

Our study also has several limitations. This was a single-arm clinical trial without control group, which affected the quality of our study design and results. All 30 smokers in our study received combined acupuncture and auricular acupressure treatments, and no controls were used for comparison. This small and relatively homogenous sample size may prevent the extrapolation of the findings and may be insufficient to generate precise results. Further large-scale, high-quality randomized controlled trials with proper comparison groups are warranted to verify our findings. In addition, we investigated the therapeutic effects of combined acupuncture and auricular acupressure for smoking cessation in moderate and heavy smokers. Future studies focusing on light and intermittent smokers would be useful since light smoking also carries substantial health risks [39]. Moreover, our follow-up period was only up to week 24. A longer follow-up period will provide more insights into the therapeutic effects of combined acupuncture and auricular acupressure for long-term smoking cessation, helping smokers to maintain a longer-term or even life-long smoke-free life. Therefore, more research is required to verify the exact role of acupuncture and auricular acupressure treatments for smoking cessation and to provide more robust and precise scientific evidence.

## Conclusions

The findings of this study indicated that acupuncture combined with auricular acupressure might help smokers to quit smoking. The intervention is safe and may help unsuccessful quit smokers to reduce their smoking and tobacco dependence. Therefore, this treatment regimen may be a valuable complementary and alternative therapeutic option for smoking cessation.

## Data Availability

The datasets used and/or analyzed during the current study are available from the corresponding author upon reasonable request.

## Abbreviations

CO: Carbon monoxide
ppm: Parts per million
FTND: Fagerström Test for Nicotine Dependence
AUTOS: Autonomy Over Smoking Scale
HAM-A: Hamilton Anxiety Rating Scale
SAS: Self-rating Anxiety Scale
PSQI: Pittsburgh Sleep Quality Index
LMM: Linear mixed-effects model
OR: Odds ratio
CI: Confidence interval
